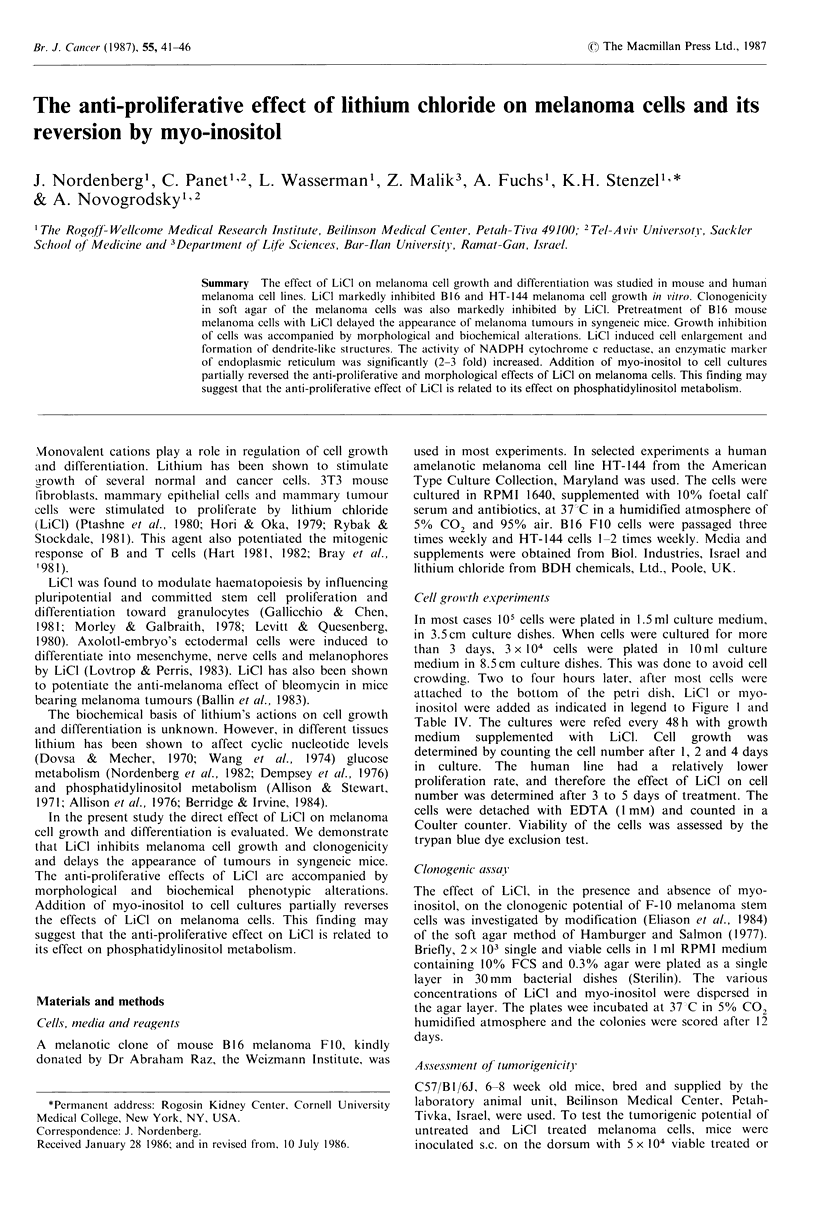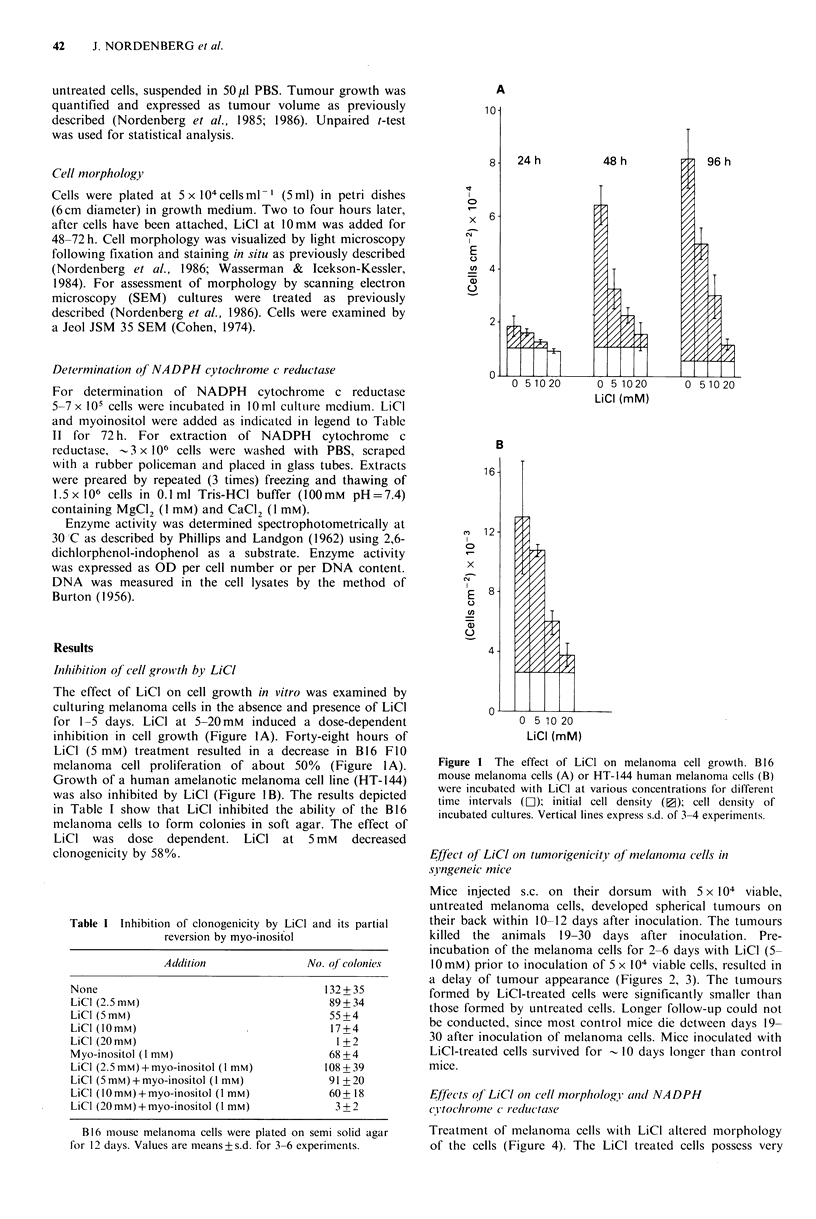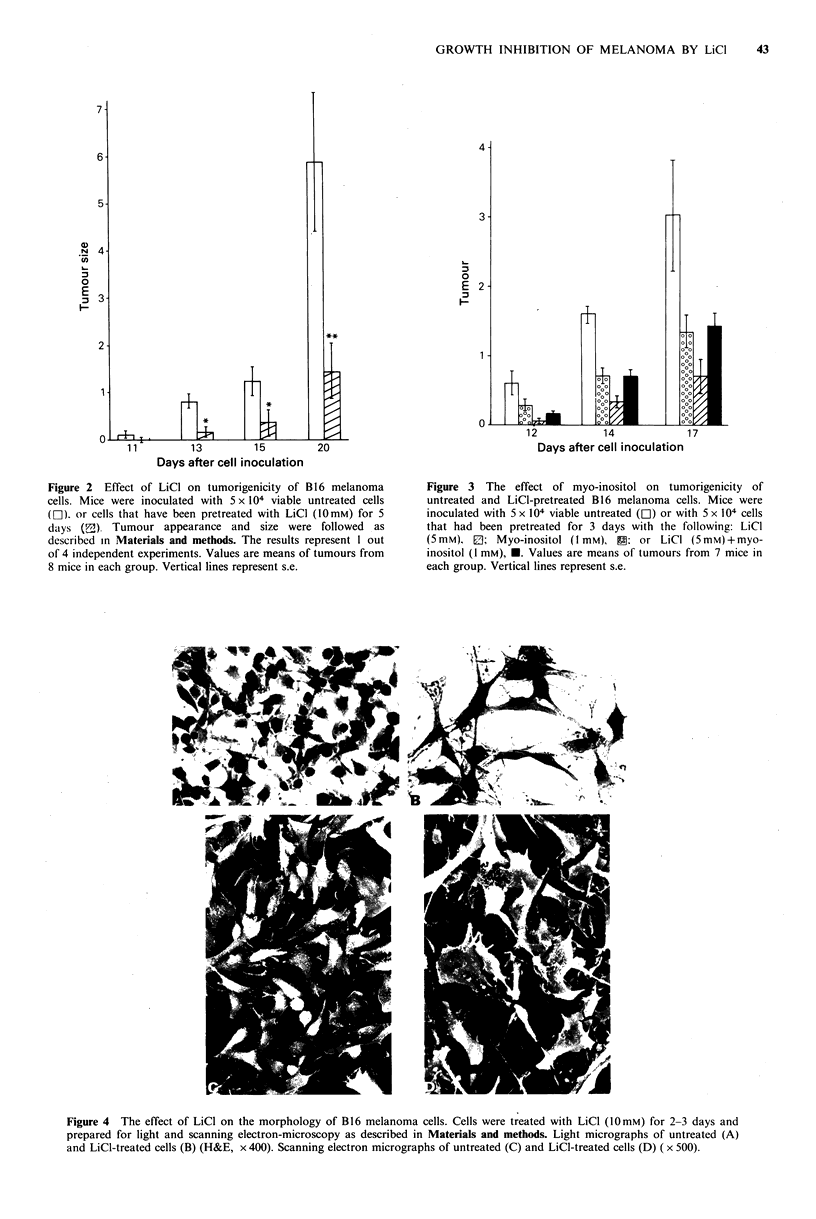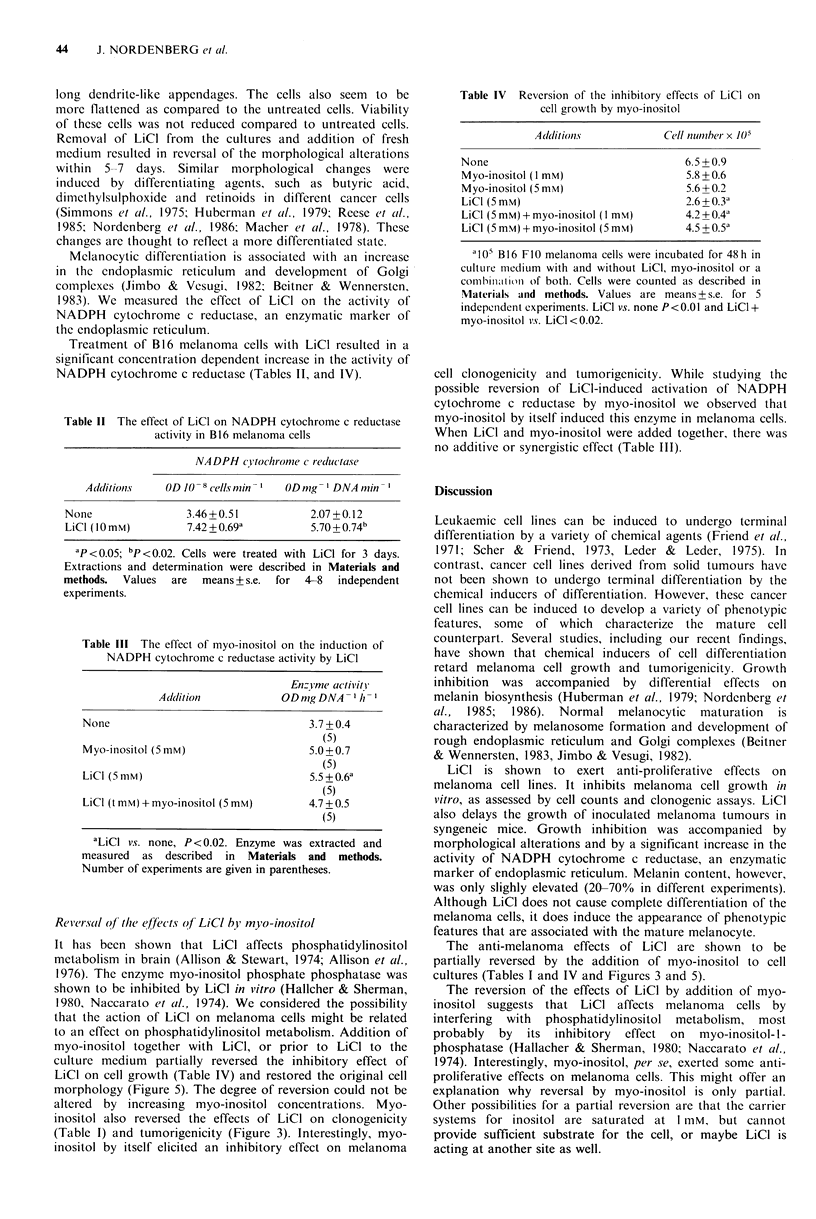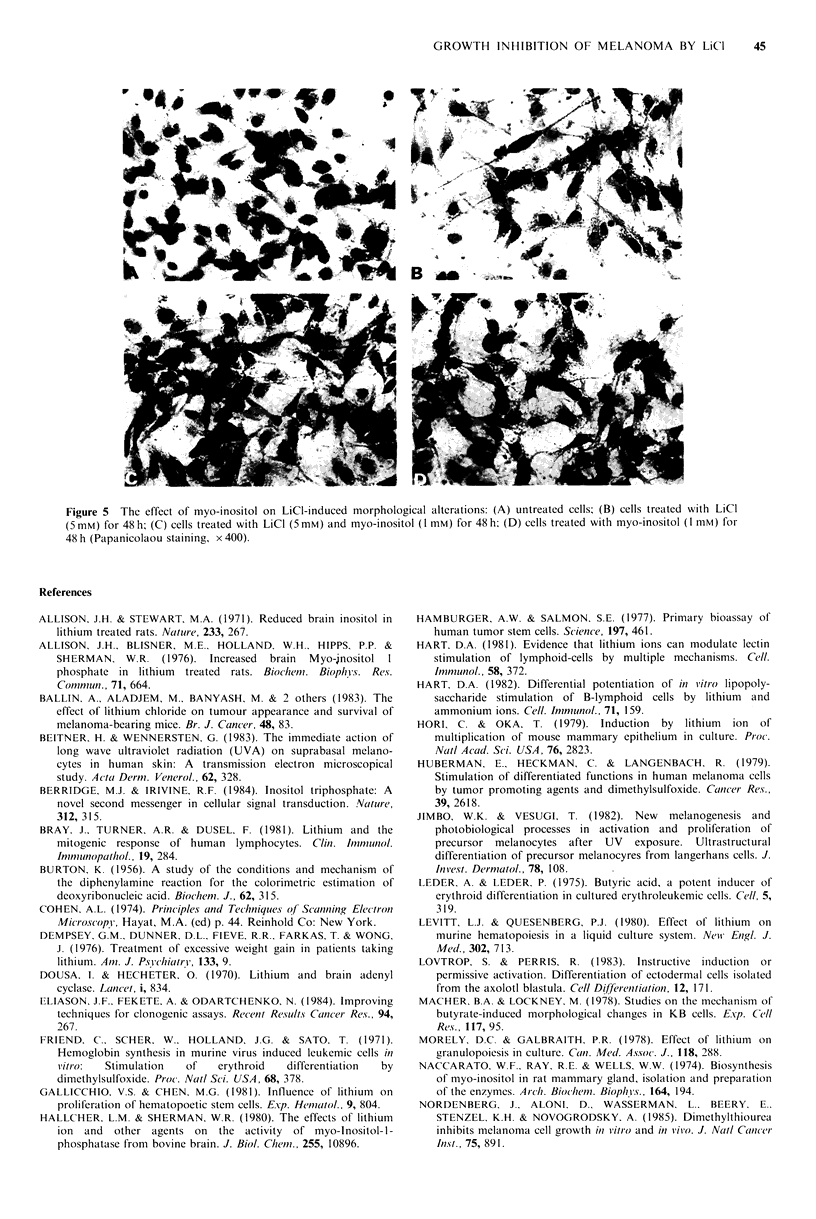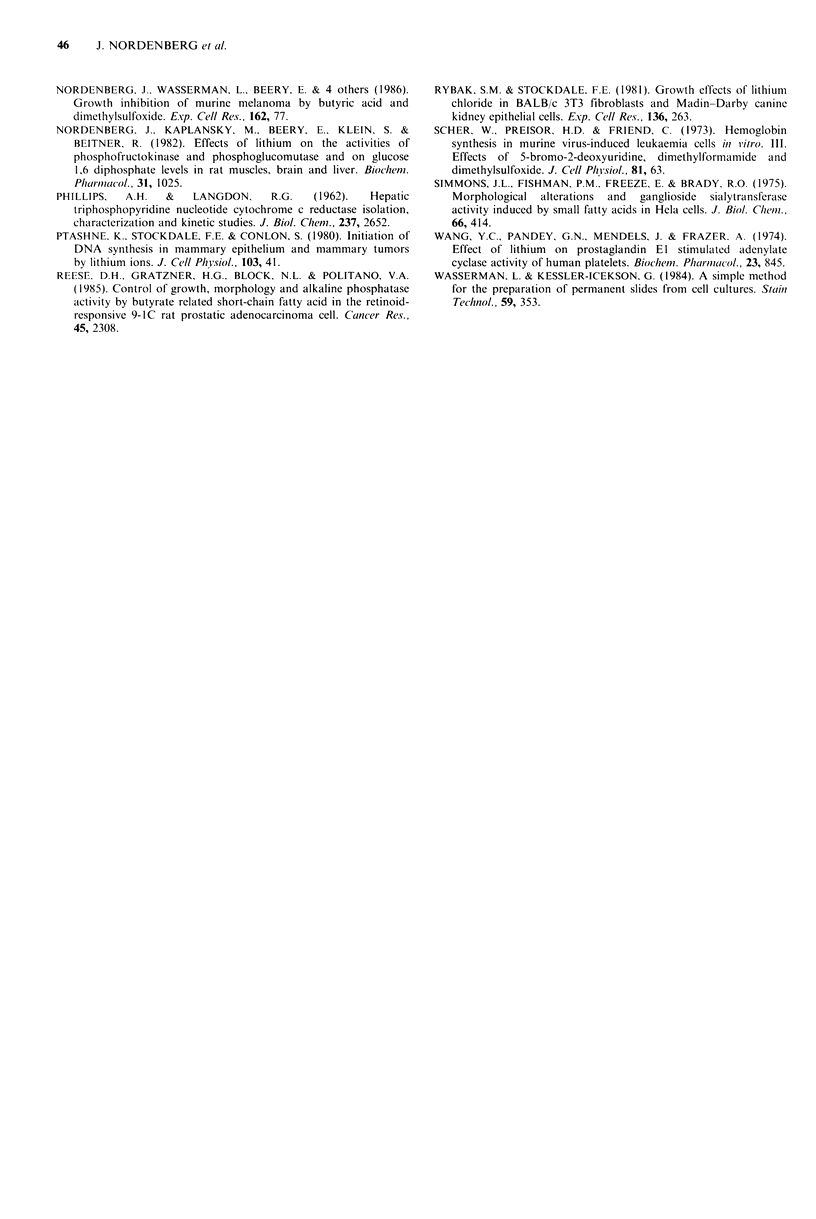# The anti-proliferative effect of lithium chloride on melanoma cells and its reversion by myo-inositol.

**DOI:** 10.1038/bjc.1987.9

**Published:** 1987-01

**Authors:** J. Nordenberg, C. Panet, L. Wasserman, Z. Malik, A. Fuchs, K. H. Stenzel, A. Novogrodsky

## Abstract

**Images:**


Br. J. Cancer (1987), 55, 41–46 © The Macmillan Press Ltd., 1987

### The anti-proliferative effect of lithium chloride on melanoma cells and its reversion by myo-inositol

J. Nordenberg[1], C. Panet[1,2], L. Wasserman[1], Z. Malik[3], A. Fuchs[1], K.H. Stenzel[1,*] & A. Novogrodsky[1,2]

[1]*The Rogoff-Wellcome Medical Research Institute, Beilinson Medical Center, Petah-Tiva 49100;* [2]*Tel-Aviv Universoty, Sackler School of Medicine and* [3]*Department of Life Sciences, Bar-Ilan University, Ramat-Gan, Israel.*

**Summary** The effect of LiCl on melanoma cell growth and differentiation was studied in mouse and human melanoma cell lines. LiCl markedly inhibited B16 and HT-144 melanoma cell growth *in vitro*. Clonogenicity in soft agar of the melanoma cells was also markedly inhibited by LiCl. Pretreatment of B16 mouse melanoma cells with LiCl delayed the appearance of melanoma tumours in syngeneic mice. Growth inhibition of cells was accompanied by morphological and biochemical alterations. LiCl induced cell enlargement and formation of dendrite-like structures. The activity of NADPH cytochrome c reductase, an enzymatic marker of endoplasmic reticulum was significantly (2–3 fold) increased. Addition of myo-inositol to cell cultures partially reversed the anti-proliferative and morphological effects of LiCl on melanoma cells. This finding may suggest that the anti-proliferative effect of LiCl is related to its effect on phosphatidylinositol metabolism.

Monovalent cations play a role in regulation of cell growth and differentiation. Lithium has been shown to stimulate growth of several normal and cancer cells. 3T3 mouse fibroblasts, mammary epithelial cells and mammary tumour cells were stimulated to proliferate by lithium chloride (LiCl) (Ptashne *et al.*, 1980; Hori & Oka, 1979; Rybak & Stockdale, 1981). This agent also potentiated the mitogenic response of B and T cells (Hart 1981, 1982; Bray *et al.*, 1981).

LiCl was found to modulate haematopoiesis by influencing pluripotential and committed stem cell proliferation and differentiation toward granulocytes (Gallicchio & Chen, 1981; Morley & Galbraith, 1978; Levitt & Quesenberg, 1980). Axolotl-embryo's ectodermal cells were induced to differentiate into mesenchyme, nerve cells and melanophores by LiCl (Lovtrop & Perris, 1983). LiCl has also been shown to potentiate the anti-melanoma effect of bleomycin in mice bearing melanoma tumours (Ballin *et al.*, 1983).

The biochemical basis of lithium's actions on cell growth and differentiation is unknown. However, in different tissues lithium has been shown to affect cyclic nucleotide levels (Dovsa & Mecher, 1970; Wang *et al.*, 1974) glucose metabolism (Nordenberg *et al.*, 1982; Dempsey *et al.*, 1976) and phosphatidylinositol metabolism (Allison & Stewart, 1971; Allison *et al.*, 1976; Berridge & Irvine, 1984).

In the present study the direct effect of LiCl on melanoma cell growth and differentiation is evaluated. We demonstrate that LiCl inhibits melanoma cell growth and clonogenicity and delays the appearance of tumours in syngeneic mice. The anti-proliferative effects of LiCl are accompanied by morphological and biochemical phenotypic alterations. Addition of myo-inositol to cell cultures partially reverses the effects of LiCl on melanoma cells. This finding may suggest that the anti-proliferative effect on LiCl is related to its effect on phosphatidylinositol metabolism.

### Materials and methods

### *Cells, media and reagents*

A melanotic clone of mouse B16 melanoma F10, kindly donated by Dr Abraham Raz, the Weizmann Institute, was used in most experiments. In selected experiments a human amelanotic melanoma cell line HT-144 from the American Type Culture Collection, Maryland was used. The cells were cultured in RPMI 1640, supplemented with 10% foetal calf serum and antibiotics, at 37°C in a humidified atmosphere of 5% $CO_2$ and 95% air. B16 F10 cells were passaged three times weekly and HT-144 cells 1–2 times weekly. Media and supplements were obtained from Biol. Industries, Israel and lithium chloride from BDH chemicals, Ltd., Poole, UK.

### *Cell growth experiments*

In most cases $10^5$ cells were plated in 1.5 ml culture medium, in 3.5 cm culture dishes. When cells were cultured for more than 3 days, $3\times10^4$ cells were plated in 10 ml culture medium in 8.5 cm culture dishes. This was done to avoid cell crowding. Two to four hours later, after most cells were attached to the bottom of the petri dish, LiCl or myo-inositol were added as indicated in legend to Figure 1 and Table IV. The cultures were refed every 48 h with growth medium supplemented with LiCl. Cell growth was determined by counting the cell number after 1, 2 and 4 days in culture. The human line had a relatively lower proliferation rate, and therefore the effect of LiCl on cell number was determined after 3 to 5 days of treatment. The cells were detached with EDTA (1 mM) and counted in a Coulter counter. Viability of the cells was assessed by the trypan blue dye exclusion test.

### *Clonogenic assay*

The effect of LiCl, in the presence and absence of myo-inositol, on the clonogenic potential of F-10 melanoma stem cells was investigated by modification (Eliason *et al.*, 1984) of the soft agar method of Hamburger and Salmon (1977). Briefly, $2\times10^3$ single and viable cells in 1 ml RPMI medium containing 10% FCS and 0.3% agar were plated as a single layer in 30 mm bacterial dishes (Sterilin). The various concentrations of LiCl and myo-inositol were dispersed in the agar layer. The plates wee incubated at 37°C in 5% $CO_2$ humidified atmosphere and the colonies were scored after 12 days.

### *Assessment of tumorigenicity*

C57/Bl/6J, 6–8 week old mice, bred and supplied by the laboratory animal unit, Beilinson Medical Center, Petah-Tivka, Israel, were used. To test the tumorigenic potential of untreated and LiCl treated melanoma cells, mice were inoculated s.c. on the dorsum with $5\times10^4$ viable treated or

*Permanent address: Rogosin Kidney Center, Cornell University Medical College, New York, NY, USA.
Correspondence: J. Nordenberg.
Received January 28 1986; and in revised from, 10 July 1986.

untreated cells, suspended in 50 μl PBS. Tumour growth was quantified and expressed as tumour volume as previously described (Nordenberg *et al.*, 1985; 1986). Unpaired *t*-test was used for statistical analysis.

*Cell morphology*

Cells were plated at $5 \times 10^4$ cells ml$^{-1}$ (5 ml) in petri dishes (6 cm diameter) in growth medium. Two to four hours later, after cells have been attached, LiCl at 10 mM was added for 48–72 h. Cell morphology was visualized by light microscopy following fixation and staining *in situ* as previously described (Nordenberg *et al.*, 1986; Wasserman & Icekson-Kessler, 1984). For assessment of morphology by scanning electron microscopy (SEM) cultures were treated as previously described (Nordenberg *et al.*, 1986). Cells were examined by a Jeol JSM 35 SEM (Cohen, 1974).

*Determination of NADPH cytochrome c reductase*

For determination of NADPH cytochrome c reductase $5–7 \times 10^5$ cells were incubated in 10 ml culture medium. LiCl and myoinositol were added as indicated in legend to Table II for 72 h. For extraction of NADPH cytochrome c reductase, $\sim 3 \times 10^6$ cells were washed with PBS, scraped with a rubber policeman and placed in glass tubes. Extracts were preared by repeated (3 times) freezing and thawing of $1.5 \times 10^6$ cells in 0.1 ml Tris-HCl buffer (100 mM pH = 7.4) containing $MgCl_2$ (1 mM) and $CaCl_2$ (1 mM).

Enzyme activity was determined spectrophotometrically at 30°C as described by Phillips and Landgon (1962) using 2,6-dichlorphenol-indophenol as a substrate. Enzyme activity was expressed as OD per cell number or per DNA content. DNA was measured in the cell lysates by the method of Burton (1956).

### Results

*Inhibition of cell growth by LiCl*

The effect of LiCl on cell growth *in vitro* was examined by culturing melanoma cells in the absence and presence of LiCl for 1-5 days. LiCl at 5–20 mM induced a dose-dependent inhibition in cell growth (Figure 1A). Forty-eight hours of LiCl (5 mM) treatment resulted in a decrease in B16 F10 melanoma cell proliferation of about 50% (Figure 1A). Growth of a human amelanotic melanoma cell line (HT-144) was also inhibited by LiCl (Figure 1B). The results depicted in Table I show that LiCl inhibited the ability of the B16 melanoma cells to form colonies in soft agar. The effect of LiCl was dose dependent. LiCl at 5 mM decreased clonogenicity by 58%.

**Table I** Inhibition of clonogenicity by LiCl and its partial reversion by myo-inositol

| *Addition* | *No. of colonies* |
|---|---|
| None | 132±35 |
| LiCl (2.5 mM) | 89±34 |
| LiCl (5 mM) | 55±4 |
| LiCl (10 mM) | 17±4 |
| LiCl (20 mM) | 1±2 |
| Myo-inositol (1 mM) | 68±4 |
| LiCl (2.5 mM)+myo-inositol (1 mM) | 108±39 |
| LiCl (5 mM)+myo-inositol (1 mM) | 91±20 |
| LiCl (10 mM)+myo-inositol (1 mM) | 60±18 |
| LiCl (20 mM)+myo-inositol (1 mM) | 3±2 |

B16 mouse melanoma cells were plated on semi solid agar for 12 days. Values are means±s.d. for 3–6 experiments.

**Figure 1** The effect of LiCl on melanoma cell growth. B16 mouse melanoma cells (A) or HT-144 human melanoma cells (B) were incubated with LiCl at various concentrations for different time intervals (□); initial cell density (▨); cell density of incubated cultures. Vertical lines express s.d. of 3–4 experiments.

*Effect of LiCl on tumorigenicity of melanoma cells in syngeneic mice*

Mice injected s.c. on their dorsum with $5 \times 10^4$ viable, untreated melanoma cells, developed spherical tumours on their back within 10–12 days after inoculation. The tumours killed the animals 19–30 days after inoculation. Pre-incubation of the melanoma cells for 2–6 days with LiCl (5–10 mM) prior to inoculation of $5 \times 10^4$ viable cells, resulted in a delay of tumour appearance (Figures 2, 3). The tumours formed by LiCl-treated cells were significantly smaller than those formed by untreated cells. Longer follow-up could not be conducted, since most control mice die detween days 19–30 after inoculation of melanoma cells. Mice inoculated with LiCl-treated cells survived for $\sim 10$ days longer than control mice.

*Effects of LiCl on cell morphology and NADPH cytochrome c reductase*

Treatment of melanoma cells with LiCl altered morphology of the cells (Figure 4). The LiCl treated cells possess very

**Figure 2** Effect of LiCl on tumorigenicity of B16 melanoma cells. Mice were inoculated with $5 \times 10^4$ viable untreated cells (□), or cells that have been pretreated with LiCl (10 mM) for 5 days (▨). Tumour appearance and size were followed as described in **Materials and methods.** The results represent 1 out of 4 independent experiments. Values are means of tumours from 8 mice in each group. Vertical lines represent s.e.

**Figure 3** The effect of myo-inositol on tumorigenicity of untreated and LiCl-pretreated B16 melanoma cells. Mice were inoculated with $5 \times 10^4$ viable untreated (□) or with $5 \times 10^4$ cells that had been pretreated for 3 days with the following: LiCl (5 mM), ▨; Myo-inositol (1 mM), ▩; or LiCl (5 mM) + myo-inositol (1 mM), ■. Values are means of tumours from 7 mice in each group. Vertical lines represent s.e.

**Figure 4** The effect of LiCl on the morphology of B16 melanoma cells. Cells were treated with LiCl (10 mM) for 2–3 days and prepared for light and scanning electron-microscopy as described in **Materials and methods.** Light micrographs of untreated (A) and LiCl-treated cells (B) (H&E, ×400). Scanning electron micrographs of untreated (C) and LiCl-treated cells (D) (×500).

long dendrite-like appendages. The cells also seem to be more flattened as compared to the untreated cells. Viability of these cells was not reduced compared to untreated cells. Removal of LiCl from the cultures and addition of fresh medium resulted in reversal of the morphological alterations within 5–7 days. Similar morphological changes were induced by differentiating agents, such as butyric acid, dimethylsulphoxide and retinoids in different cancer cells (Simmons *et al.*, 1975; Huberman *et al.*, 1979; Reese *et al.*, 1985; Nordenberg *et al.*, 1986; Macher *et al.*, 1978). These changes are thought to reflect a more differentiated state.

Melanocytic differentiation is associated with an increase in the endoplasmic reticulum and development of Golgi complexes (Jimbo & Vesugi, 1982; Beitner & Wennersten, 1983). We measured the effect of LiCl on the activity of NADPH cytochrome c reductase, an enzymatic marker of the endoplasmic reticulum.

Treatment of B16 melanoma cells with LiCl resulted in a significant concentration dependent increase in the activity of NADPH cytochrome c reductase (Tables II, and IV).

**Table II** The effect of LiCl on NADPH cytochrome c reductase activity in B16 melanoma cells

| | NADPH cytochrome c reductase | |
|---|---|---|
| *Additions* | *OD $10^{-8}$ cells $min^{-1}$* | *OD $mg^{-1}$ DNA $min^{-1}$* |
| None | 3.46±0.51 | 2.07±0.12 |
| LiCl (10 mM) | 7.42±0.69[a] | 5.70±0.74[b] |

[a]$P<0.05$; [b]$P<0.02$. Cells were treated with LiCl for 3 days. Extractions and determination were described in **Materials and methods.** Values are means±s.e. for 4–8 independent experiments.

**Table III** The effect of myo-inositol on the induction of NADPH cytochrome c reductase activity by LiCl

| *Addition* | *Enzyme activity OD mg $DNA^{-1}$ $h^{-1}$* |
|---|---|
| None | 3.7±0.4 (5) |
| Myo-inositol (5 mM) | 5.0±0.7 (5) |
| LiCl (5 mM) | 5.5±0.6[a] (5) |
| LiCl (t mM) + myo-inositol (5 mM) | 4.7±0.5 (5) |

[a]LiCl *vs.* none, $P<0.02$. Enzyme was extracted and measured as described in **Materials and methods.** Number of experiments are given in parentheses.

*Reversal of the effects of LiCl by myo-inositol*

It has been shown that LiCl affects phosphatidylinositol metabolism in brain (Allison & Stewart, 1974; Allison *et al.*, 1976). The enzyme myo-inositol phosphate phosphatase was shown to be inhibited by LiCl *in vitro* (Hallcher & Sherman, 1980, Naccarato *et al.*, 1974). We considered the possibility that the action of LiCl on melanoma cells might be related to an effect on phosphatidylinositol metabolism. Addition of myo-inositol together with LiCl, or prior to LiCl to the culture medium partially reversed the inhibitory effect of LiCl on cell growth (Table IV) and restored the original cell morphology (Figure 5). The degree of reversion could not be altered by increasing myo-inositol concentrations. Myo-inositol also reversed the effects of LiCl on clonogenicity (Table I) and tumorigenicity (Figure 3). Interestingly, myo-inositol by itself elicited an inhibitory effect on melanoma

**Table IV** Reversion of the inhibitory effects of LiCl on cell growth by myo-inositol

| *Additions* | *Cell number × $10^5$* |
|---|---|
| None | 6.5±0.9 |
| Myo-inositol (1 mM) | 5.8±0.6 |
| Myo-inositol (5 mM) | 5.6±0.2 |
| LiCl (5 mM) | 2.6±0.3[a] |
| LiCl (5 mM) + myo-inositol (1 mM) | 4.2±0.4[a] |
| LiCl (5 mM) + myo-inositol (5 mM) | 4.5±0.5[a] |

[a]$10^5$ B16 F10 melanoma cells were incubated for 48 h in culture medium with and without LiCl, myo-inositol or a combination of both. Cells were counted as described in **Materials and methods.** Values are means±s.e. for 5 independent experiments. LiCl *vs.* none $P<0.01$ and LiCl + myo-inositol *vs.* LiCl < 0.02.

cell clonogenicity and tumorigenicity. While studying the possible reversion of LiCl-induced activation of NADPH cytochrome c reductase by myo-inositol we observed that myo-inositol by itself induced this enzyme in melanoma cells. When LiCl and myo-inositol were added together, there was no additive or synergistic effect (Table III).

### Discussion

Leukaemic cell lines can be induced to undergo terminal differentiation by a variety of chemical agents (Friend *et al.*, 1971; Scher & Friend, 1973, Leder & Leder, 1975). In contrast, cancer cell lines derived from solid tumours have not been shown to undergo terminal differentiation by the chemical inducers of differentiation. However, these cancer cell lines can be induced to develop a variety of phenotypic features, some of which characterize the mature cell counterpart. Several studies, including our recent findings, have shown that chemical inducers of cell differentiation retard melanoma cell growth and tumorigenicity. Growth inhibition was accompanied by differential effects on melanin biosynthesis (Huberman *et al.*, 1979; Nordenberg *et al.*, 1985; 1986). Normal melanocytic maturation is characterized by melanosome formation and development of rough endoplasmic reticulum and Golgi complexes (Beitner & Wennersten, 1983, Jimbo & Vesugi, 1982).

LiCl is shown to exert anti-proliferative effects on melanoma cell lines. It inhibits melanoma cell growth *in vitro*, as assessed by cell counts and clonogenic assays. LiCl also delays the growth of inoculated melanoma tumours in syngeneic mice. Growth inhibition was accompanied by morphological alterations and by a significant increase in the activity of NADPH cytochrome c reductase, an enzymatic marker of endoplasmic reticulum. Melanin content, however, was only slighly elevated (20–70% in different experiments). Although LiCl does not cause complete differentiation of the melanoma cells, it does induce the appearance of phenotypic features that are associated with the mature melanocyte.

The anti-melanoma effects of LiCl are shown to be partially reversed by the addition of myo-inositol to cell cultures (Tables I and IV and Figures 3 and 5).

The reversion of the effects of LiCl by addition of myo-inositol suggests that LiCl affects melanoma cells by interfering with phosphatidylinositol metabolism, most probably by its inhibitory effect on myo-inositol-1-phosphatase (Hallacher & Sherman, 1980; Naccarato *et al.*, 1974). Interestingly, myo-inositol, *per se*, exerted some anti-proliferative effects on melanoma cells. This might offer an explanation why reversal by myo-inositol is only partial. Other possibilities for a partial reversion are that the carrier systems for inositol are saturated at 1 mM, but cannot provide sufficient substrate for the cell, or maybe LiCl is acting at another site as well.

**Figure 5** The effect of myo-inositol on LiCl-induced morphological alterations: (A) untreated cells; (B) cells treated with LiCl (5 mM) for 48 h; (C) cells treated with LiCl (5 mM) and myo-inositol (1 mM) for 48 h; (D) cells treated with myo-inositol (1 mM) for 48 h (Papanicolaou staining, ×400).

### References

ALLISON, J.H. & STEWART, M.A. (1971). Reduced brain inositol in lithium treated rats. *Nature*, **233**, 267.

ALLISON, J.H., BLISNER, M.E., HOLLAND, W.H., HIPPS, P.P. & SHERMAN, W.R. (1976). Increased brain Myo-inositol 1 phosphate in lithium treated rats. *Biochem. Biophys. Res. Commun.*, **71**, 664.

BALLIN, A., ALADJEM, M., BANYASH, M. & 2 others (1983). The effect of lithium chloride on tumour appearance and survival of melanoma-bearing mice. *Br. J. Cancer*, **48**, 83.

BEITNER, H. & WENNERSTEN, G. (1983). The immediate action of long wave ultraviolet radiation (UVA) on suprabasal melanocytes in human skin: A transmission electron microscopical study. *Acta Derm. Venerol.*, **62**, 328.

BERRIDGE, M.J. & IRIVINE, R.F. (1984). Inositol triphosphate: A novel second messenger in cellular signal transduction. *Nature*, **312**, 315.

BRAY, J., TURNER, A.R. & DUSEL, F. (1981). Lithium and the mitogenic response of human lymphocytes. *Clin. Immunol. Immunopathol.*, **19**, 284.

BURTON, K. (1956). A study of the conditions and mechanism of the diphenylamine reaction for the colorimetric estimation of deoxyribonucleic acid. *Biochem. J.*, **62**, 315.

COHEN, A.L. (1974). *Principles and Techniques of Scanning Electron Microscopy*, Hayat, M.A. (ed) p. 44. Reinhold Co: New York.

DEMPSEY, G.M., DUNNER, D.L., FIEVE, R.R., FARKAS, T. & WONG, J. (1976). Treatment of excessive weight gain in patients taking lithium. *Am. J. Psychiatry*, **133**, 9.

DOUSA, I. & HECHETER, O. (1970). Lithium and brain adenyl cyclase. *Lancet*, **i**, 834.

ELIASON, J.F., FEKETE, A. & ODARTCHENKO, N. (1984). Improving techniques for clonogenic assays. *Recent Results Cancer Res.*, **94**, 267.

FRIEND, C., SCHER, W., HOLLAND, J.G. & SATO, T. (1971). Hemoglobin synthesis in murine virus induced leukemic cells *in vitro*: Stimulation of erythroid differentiation by dimethylsulfoxide. *Proc. Natl Sci. USA*, **68**, 378.

GALLICCHIO, V.S. & CHEN, M.G (1981). Influence of lithium on proliferation of hematopoetic stem cells. *Exp. Hematol.*, **9**, 804.

HALLCHER, L.M. & SHERMAN, W.R. (1980). The effects of lithium ion and other agents on the activity of myo-Inositol-1-phosphatase from bovine brain. *J. Biol. Chem.*, **255**, 10896.

HAMBURGER, A.W. & SALMON, S.E. (1977). Primary bioassay of human tumor stem cells. *Science*, **197**, 461.

HART, D.A. (1981). Evidence that lithium ions can modulate lectin stimulation of lymphoid-cells by multiple mechanisms. *Cell. Immunol.*, **58**, 372.

HART, D.A. (1982). Differential potentiation of *in vitro* lipopolysaccharide stimulation of B-lymphoid cells by lithium and ammonium ions. *Cell. Immunol.*, **71**, 159.

HORI, C. & OKA, T. (1979). Induction by lithium ion of multiplication of mouse mammary epithelium in culture. *Proc. Natl Acad. Sci. USA*, **76**, 2823.

HUBERMAN, E., HECKMAN, C. & LANGENBACH, R. (1979). Stimulation of differentiated functions in human melanoma cells by tumor promoting agents and dimethylsulfoxide. *Cancer Res.*, **39**, 2618.

JIMBO, W.K. & VESUGI, T. (1982). New melanogenesis and photobiological processes in activation and proliferation of precursor melanocytes after UV exposure. Ultrastructural differentiation of precursor melanocyres from langerhans cells. *J. Invest. Dermatol.*, **78**, 108.

LEDER, A. & LEDER, P. (1975). Butyric acid, a potent inducer of erythroid differentiation in cultured erythroleukemic cells. *Cell*, **5**, 319.

LEVITT, L.J. & QUESENBERG, P.J. (1980). Effect of lithium on murine hematopoiesis in a liquid culture system. *New Engl. J. Med.*, **302**, 713.

LOVTROP, S. & PERRIS, R. (1983). Instructive induction or permissive activation. Differentiation of ectodermal cells isolated from the axolotl blastula. *Cell Differentiation*, **12**, 171.

MACHER, B.A. & LOCKNEY, M. (1978). Studies on the mechanism of butyrate-induced morphological changes in KB cells. *Exp. Cell Res.*, **117**, 95.

MORELY, D.C. & GALBRAITH, P.R. (1978). Effect of lithium on granulopoiesis in culture. *Can. Med. Assoc. J.*, **118**, 288.

NACCARATO, W.F., RAY, R.E. & WELLS, W.W. (1974). Biosynthesis of myo-inositol in rat mammary gland, isolation and preparation of the enzymes. *Arch. Biochem. Biophys.*, **164**, 194.

NORDENBERG, J., ALONI, D., WASSERMAN, L., BEERY, E., STENZEL, K.H. & NOVOGRODSKY, A. (1985). Dimethylthiourea inhibits melanoma cell growth *in vitro* and *in vivo*. *J. Natl Cancer Inst.*, **75**, 891.

NORDENBERG, J., WASSERMAN, L., BEERY, E. & 4 others (1986). Growth inhibition of murine melanoma by butyric acid and dimethylsulfoxide. *Exp. Cell Res.*, **162,** 77.

NORDENBERG, J., KAPLANSKY, M., BEERY, E., KLEIN, S. & BEITNER, R. (1982). Effects of lithium on the activities of phosphofructokinase and phosphoglucomutase and on glucose 1,6 diphosphate levels in rat muscles, brain and liver. *Biochem. Pharmacol.*, **31,** 1025.

PHILLIPS, A.H. & LANGDON, R.G. (1962). Hepatic triphosphopyridine nucleotide cytochrome c reductase isolation, characterization and kinetic studies. *J. Biol. Chem.*, **237,** 2652.

PTASHNE, K., STOCKDALE, F.E. & CONLON, S. (1980). Initiation of DNA synthesis in mammary epithelium and mammary tumors by lithium ions. *J. Cell Physiol.*, **103,** 41.

REESE, D.H., GRATZNER, H.G., BLOCK, N.L. & POLITANO, V.A. (1985). Control of growth, morphology and alkaline phosphatase activity by butyrate related short-chain fatty acid in the retinoid-responsive 9-1C rat prostatic adenocarcinoma cell. *Cancer Res.*, **45,** 2308.

RYBAK, S.M. & STOCKDALE, F.E. (1981). Growth effects of lithium chloride in BALB/c 3T3 fibroblasts and Madin–Darby canine kidney epithelial cells. *Exp. Cell Res.*, **136,** 263.

SCHER, W., PREISOR, H.D. & FRIEND, C. (1973). Hemoglobin synthesis in murine virus-induced leukaemia cells *in vitro*. III. Effects of 5-bromo-2-deoxyuridine, dimethylformamide and dimethylsulfoxide. *J. Cell Physiol.*, **81,** 63.

SIMMONS, J.L., FISHMAN, P.M., FREEZE, E. & BRADY, R.O. (1975). Morphological alterations and ganglioside sialytransferase activity induced by small fatty acids in Hela cells. *J. Biol. Chem.*, **66,** 414.

WANG, Y.C., PANDEY, G.N., MENDELS, J. & FRAZER, A. (1974). Effect of lithium on prostaglandin E1 stimulated adenylate cyclase activity of human platelets. *Biochem. Pharmacol.*, **23,** 845.

WASSERMAN, L. & KESSLER-ICEKSON, G. (1984). A simple method for the preparation of permanent slides from cell cultures. *Stain Technol.*, **59,** 353.